# The Effect of Physical Exercise on Oxidation Capacity and Utero-Placental Circulation in Pregnancies with Gestational Diabetes Mellitus and Uncomplicated Pregnancies, a Pilot Study

**DOI:** 10.3390/diagnostics12071732

**Published:** 2022-07-16

**Authors:** Christos Chatzakis, Alexandros Sotiriadis, Ioannis G. Fatouros, Athanasios Z. Jamurtas, Chariklia K. Deli, Maria Papagianni, Konstantinos Dinas, George Mastorakos

**Affiliations:** 1 2nd Department of Obstetrics and Gynecology, School of Medicine, Aristotle University of Thessaloniki, 54642 Thessaloniki, Greece; cchatzak@auth.gr (C.C.); alesoti@auth.gr (A.S.); dinas@auth.gr (K.D.); 2 Department of Physical Education and Sport Sciences, School of Physical Education, Sport Science & Dietetics, University of Thessaly, 42100 Trikala, Greece; ifatouros@pe.uth.gr (I.G.F.); ajamurt@pe.uth.gr (A.Z.J.); delixar@uth.gr (C.K.D.); mpapag@uth.gr (M.P.); 3 Endocrine Unit of Aretaieion Hospital, Medical School, National and Capodistrian University of Athens, 11528 Athens, Greece

**Keywords:** exercise bout, gestational diabetes mellitus, oxidation capacity, utero-placental circulation

## Abstract

Background: Gestational diabetes mellitus (GDM) is associated with aggravated oxidation capacity and utero-placental circulation, while aerobic exercise could improve both. The study aims to assess oxidation capacity and utero-placental circulation before and after a bout of aerobic exercise in GDM and uncomplicated pregnancies.; Methods: In this cross-sectional study, women with GDM (GDMs) and women with uncomplicated pregnancies(controls), underwent 30 min of moderate intensity cycling. Total antioxidant capacity (TAC), catalase activity (CAT), reduced glutathione (GSH), Uterine Arteries (UtA PI) and Umbilical Artery (UmA PI) pulsatility indexes were estimated prior-to, immediately after and one hour after exercise; Results: In each group, 25 pregnant women were included. In both groups, between prior-to and immediately after exercise, TAC and CAT increased, while GSH decreased, (*p* < 0.001). In GDMs, CAT was lower than controls at any time point (*p* < 0.05), while in GDMs delta(Δ) CAT (prior-to and immediately after exercise) was lower than controls (*p* = 0.003). In GDMs, UtA PI centiles decreased between prior-to and either immediately or one hour after exercise, while they did not change in controls. In GDMs, pre-conceptional BMI and weight gain predicted negatively ΔTAC (prior-to to one hour after exercise); Conclusions: Moderate intensity exercise bout improves oxidation capacity in GDM and uncomplicated pregnancies, although at a lesser extent in the former. Exercise leads to decreased UtA arteries resistance in women with GDM.

## 1. Introduction

In uncomplicated pregnancies the body of the mother undergoes numerous physiological changes which contribute to an environment appropriate for the developing fetus [1]. Enhanced metabolism, increased insulin resistance, high consumption of oxygen and utilization of fatty acids which accompany pregnancy, result in increased production of Reactive Oxygen Species (ROS) [2]. At the same time, anti-oxidation mechanisms intensify their efficiency during the second and third trimesters [3].

Gestational diabetes mellitus (GDM) is defined as glucose intolerance first diagnosed during pregnancy. It is accompanied by inadequate pancreatic function, insufficient to overcome the developing pregnancy-related “physiologic” insulin resistance [4]. In GDM, the resulting hyperglycemic environment is associated with overproduction of ROS and impairment of anti-oxidation mechanisms [5,6]. Increased insulin resistance and overproduction of ROS lead to uncoupling of endothelial nitric oxide (NO) synthase, which is responsible for the regulation of vascular endothelial cell function via NO production [7]. The resulting decreased synthesis and bioavailability of NO is followed by endothelial dysfunction in maternal and placental vessels [8]. Moreover, combination of insulin resistance and increased glucose concentration leads to endothelial dysfunction in pregnancies with GDM, which is evident even from the first trimester [9,10].

Regular mild to moderate intensity physical exercise during pregnancy is highly recommended, as it is associated with various beneficial effects to the mothers and their fetuses [11,12]. Off pregnancy, it is well established that aerobic exercise improves endothelial function, by increasing NO synthesis in both the endothelium and the smooth muscle layer of the vessels [13,14]. Moreover, aerobic exercise improves oxidation capacity by augmenting the antioxidant mechanisms [15]. More specifically, the transient increase in pro-oxidation markers during exercise, induces a compensatory increase in the activity of antioxidant mechanisms [16].

The aim of the present study was to assess anti- oxidation parameters as well as utero-placental circulation before and after a bout of aerobic exercise of moderate intensity in women with GDM as compared to women with uncomplicated pregnancies.

## 2. Materials and Methods

### 2.1. Study Design

In this pilot cross-sectional study women with GDM and women with uncomplicated pregnancies were recruited between February 2020 and February 2021 from pregnant women consulting at the outpatient clinic of the Second Department of Obstetrics and Gynecology of the Aristotle University of Thessaloniki, for the follow-up of their pregnancies.

### 2.2. Participants

All pregnant women at 24–28 weeks of gestation underwent screening for GDM with a 75 g two-hour oral glucose tolerance Test (OGTT). Gestational diabetes mellitus was diagnosed as per the International Association of Diabetes and Pregnancy Study Groups (IADPSG) criteria (one blood glucose value equal or greater than either 92 mg/dL, or 180 mg/dL, or 153 mg/dL, at either fasting, or 60 min or 120 min, respectively, after consumption of 75 g of glucose secured positive diagnosis) [17].

The aim of this study was to identify potential defects in oxidation capacity and utero-placental circulation in GDM pregnancies by employing physical exercise as amplifier. To answer the formulated hypotheses GDM women should be compared to women with normal carbohydrate metabolism (controls). To reflect the latter, the control group was formed by uncomplicated pregnancies, because complications (i.e., inflammation, stress, etc.) in otherwise normal (regarding GDM) women might affect indirectly or directly the physiology of carbohydrates, oxidation capacity and utero-placental circulation. Therefore, the inclusion criteria were formed as follows: women with positive GDM diagnosis were offered to participate in the study, whereas women with negative GDM diagnosis and uncomplicated pregnancies were offered to participate in the study as controls. Women with GDM were matched with women with uncomplicated pregnancies according to pre-pregnancy BMI, maternal age and gestational age. Exclusion criteria comprised pregnancies with pre-existing diabetes mellitus (type 1 or 2), hypertensive disorders of pregnancy, chromosomally abnormal fetuses with or without structural defects, fetal growth restriction and smoking habitus.

All participants signed an informed consent form and they agreed that their anonymized data could be used for research purposes. The protocol of the study was approved by the ethical committee and the institutional review board (Bioethical Committee of the Medical School of Aristotle University of Thessaloniki, protocol code 281, 27 February 2019), in accordance with the Declaration of Helsinki.

### 2.3. Variables

At the initial visit, maternal and pregnancy characteristics were recorded, including maternal age, gestational age, maternal height, pre-pregnancy weight and BMI, and weight gain (Table 1). Physical activity status of participants was assessed by the self-reporting International Physical Activity Questionnaire (IPAQ) [18]. Subsequently all participants underwent 30 min of moderate intensity (at 60–70% of the estimated maximum heart rate for their age) cycling on a stationary bicycle (KETTLER ERGO C4 Exercise Bike, Ense-Parsit, Germany). All women used the heart rate monitor of the exercise bike during this bout of aerobic exercise to ensure moderate exercise intensity, while the rating of perceived exertion scale ranged from 12 to 14 [19]. In all participants, venous blood sampling for measurement of total antioxidant capacity (TAC), catalase activity (CAT), and reduced glutathione (GSH) and Doppler ultrasound examination of embryo-placental circulation for measurement of the pulsatility index of the maternal uterine arteries (UtA PI) and the pulsatility index of the umbilical artery (UmA PI) were performed before, immediately after and one-hour after the exercise bout. Blood was collected into EDTA tubes, or tubes containing SST-Gel and subsequently centrifuged for plasma and serum (for measurement of TAC concentration) separation, respectively. Red blood cells collected after plasma separation, were lysed, and the lysate was used for the analysis of catalase activity and GSH concentrations. Samples were stored in multiple aliquots (lysate and serum samples at −80 °C), were protected from light and auto-oxidation, and were thawed, once, before analysis.

Total antioxidant capacity, catalase, and GSH, were determined in a HITACHI, U-1900 spectrophotometer as previously described [20]. Intra- and inter- assay coefficient of variation (CV) for TAC was 3% and 3.4%, respectively, for catalase 3.8% and 8.9%, respectively, and for GSH 3.6% and 1.6%, respectively. All assays were performed in duplicate, and the mean value was recorded.

For the measurement of the Pulsatility Index of the maternal uterine arteries and umbilical artery, a General Electric Voluson S10 (GE Healthcare; Zipf, Austria) ultrasound machine with convex transducer (RAB6-RS) was used. All ultrasound examinations were performed by examiners certified for doppler assessment sonographers by Fetal Medicine Foundation (www.fetalmedicine.com (accessed on 1 June 2022)). Transformation of uterine arteries and of umbilical artery pulsatility indexes to centiles were made using the Gomez et al., and the Arduini et al., reference curves, respectively [21,22].

### 2.4. Study Size

The sample size was determined by estimating the change in TAC concentration based on a previous study, assuming 0.4 μmol/mL increase in TAC values after exercise with 80% power and 5% significance level [23]. The conventional levels of 80% power and of 5% significance, which are more frequently used in pilot studies, were employed. Power calculation resulted in 44 patients and including 10% attrition rate, the target of the study had been set at recruitment of 50 patients altogether. The sample size calculation was carried out using the GPower software 3.1 (University of Dusseldorf, Dusseldorf, Germany).

### 2.5. Statistical Methods

Continuous variables were presented as mean and standard deviation (SD), if their distribution was normal, or as medians and interquartile range values if the distribution was non-normal. Categorical variables were summarized as percentages. In continuous variables, the differences between prior-to and immediately or one hour after the exercise bout as well as between immediately after and one hour after the exercise bout were reported as delta (Δ). Quantitative variables were compared between the two groups at the different time-points by employing General Linear Models ANOVA (Non-matched) and Bonferroni post-hoc test. Repeated measures ANOVA involved one factor between patients (factor “Group” with two levels) and one factor for the repeated measures within patients (factor “time” with 3 levels). Chi-square or Fisher’s exact test were used for pairwise comparisons of proportions, as appropriate, and odds ratios (ORs) along with their 95% confidence intervals (CIs) were calculated. In all the above tests, a *p*-value of <0.05 was considered significant.

Forward stepwise linear regression analysis was employed in both groups, in order to reveal potential correlations and confounders, including maternal pre-pregnancy BMI, maternal age, maternal weight gain, parity, history of GDM and physical activity as independent variables and ΔTAC, ΔGSH, ΔCAT, ΔUtA and ΔUmA centile as dependent variables.The analyses were performed on open source software R 2.15.1 (The R Foundation for Statistical Computing, Vienna, Austria).

## 3. Results

### 3.1. Participants

**Figure 1:**
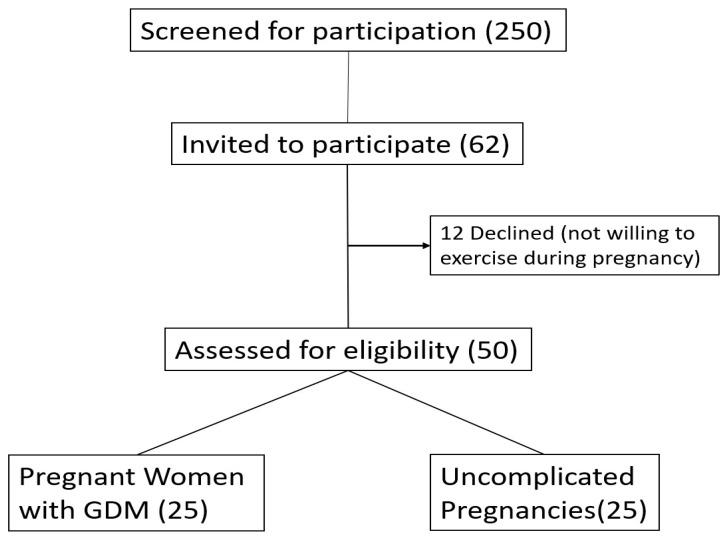
Inclusion Algorythm.

In this case, 250 pregnant women visiting consecutively the obstetric outpatient clinic of the Second Department of Obstetrics and Gynecology of Aristotle University of Thessaloniki for a regular follow-up of their pregnancy, were screened for participation in the study. Among them, 32 pregnant women were identified with GDM and were offered to participate in the study; 25 of the 32 agreed to participate in the study. Then 30 women with uncomplicated pregnancies (out of the 250 consecutively examined) were matched to the already recruited 25 GDM pregnancies regarding pre-pregnancy BMI, maternal age and gestational age and were asked to participate in the study. From them 25 agreed finally to participate in the study. Thus, 50 pregnant women agreed to participate in the study (25 with GDM and 25 with uncomplicated pregnancies) (Figure 1).

### 3.2. Descriptive Data

There were no statistically significant differences between the studied groups of pregnant women regarding BMI (before pregnancy and at the study period), weight gain, maternal age, mode of conception, parity, gestational age and physical activity. Presence of GDM in previous pregnancies differed significantly between the studied groups (four women with GDM vs. no woman with uncomplicated pregnancy, *p* = 0.043) (Table 1).

### 3.3. Main Results

In both groups, TAC mean values increased significantly from prior-to to immediately after the exercise bout (*p* < 0.001), while they did not differ between either prior-to and one hour after the exercise bout or between immediately after and one hour after the exercise bout. Prior to the exercise bout there was a trend towards decreased TAC mean values in women with GDM compared to women with uncomplicated pregnancies (*p* = 0.066). Between the two groups, TAC mean values did not differ significantly immediately after and one hour after the exercise bout. No significant differences were found regarding any ΔTAC value in the three time points, between the two groups (Table 2).

In both groups, mean values of catalase activity increased significantly from prior-to to immediately after the exercise bout (*p* < 0.001), while they did not differ between either prior-to and one hour after the exercise bout or between immediately after and one hour after the exercise bout. Mean values of catalase activity were significantly lower in women with GDM compared to women with uncomplicated pregnancies, at any time point (*p* < 0.05, respectively). Delta catalase activity between prior-to and immediately after the exercise bout was significantly lower in women with GDM compared to women with uncomplicated pregnancies (36.7 ± 32.6 vs. 66.9 ± 27.1, *p* = 0.003). No significant differences were found regarding Δcatalase activity values between prior-to and one hour after the exercise bout, between the two groups (Table 2).

In both groups, GSH mean values decreased significantly from prior-to to either immediately after (*p* < 0.001) or one hour after (*p* < 0.05) the exercise bout, while they did not differ between immediately after and one hour after the exercise bout. At immediately after the exercise bout, there were statistically significant decreased GSH mean values in women with GDM compared to women with uncomplicated pregnancies (*p* < 0.05). No significant differences were found regarding GSH mean values prior-to and one hour after the exercise bout, between the two groups. No significant differences were found regarding any ΔGSH value in the three time points, between the two groups (Table 2).

In the GDM group, mean UtA PI centiles decreased significantly from prior-to to either immediately after or one hour after the exercise bout (*p* < 0.05). Between the two groups, mean UtA PI centiles did not differ significantly prior to the exercise bout. Mean UtA PI centiles were significantly lower in women with GDM compared to women with uncomplicated pregnancies immediately after and one hour after the exercise bout (*p* < 0.01). The changes of Uta PI centiles (ΔUtA PI) between prior-to and either immediately after or one hour after the exercise bout were significantly greater in women with GDM compared to women with uncomplicated pregnancies (−16.2 ± 26.8 vs. 3.5 ± 21.3, *p* = 0.011 and −16.0 ± 23.8 vs −2.2 ± 15.3, *p* = 0.030, respectively). Mean UmA PI centiles did not differ significantly at any time point, between the two groups as well as within each group (Table 3) (Figure S1).

When forward stepwise linear regression analysis was employed to reveal potential predictors of either ΔTAC or ΔCAT or ΔGS taken as dependent variables (Δ representing the difference between either prior-to and immediately after the exercise bout or prior-to and one hour after the exercise bout) with maternal age, maternal pre-pregnancy BMI and weigh gain, parity, history of GDM and physical activity taken as independent variables. Pre-conceptional BMI and weight gain were found to be negative predictors (b = −13.8; *p* = 0.023 and b =−5.2; *p* = 0.030) of ΔTAC between prior-to and one hour after the exercise bout in the GDM group, while no significant predictors were found in women with uncomplicated pregnancies.

When forward stepwise linear regression analysis was employed to reveal potential predictors of either ΔUtA PI or ΔUmA PI taken as dependent variables (Δ representing the difference between either prior-to and immediately after the exercise bout or prior-to and one hour after the exercise bout) with maternal age, maternal pre-pregnancy BMI and weigh gain, parity, history of GDM and physical activity taken as independent variables, no significant predictors were found in either group.

## 4. Discussion

### 4.1. Main Findings

We found that, in pregnant women at the third trimester, a bout of aerobic exercise of moderate intensity (cycling) resulted in increase of TAC and catalase activity measurements in both women with GDM and women with uncomplicated pregnancies. However, in all time points, catalase activity was significantly lower in women with GDM compared to women with uncomplicated pregnancies, while its increase immediately after the bout of exercise was less pronounced in the former compared to the latter. Similarly, a trend for lower TAC measurements prior (baseline) to the bout of exercise of moderate intensity was observed in women with GDM compared to women with uncomplicated pregnancies.

### 4.2. Interpretation

To our knowledge, this is the first time that exercise in pregnancy is studied in relation to oxidation status. Previous studies in non-pregnant populations, have shown that a bout of exercise results in marked elevations of biomarkers of pro- and anti- oxidation status at rates of variable magnitude [23,24,25]. Free radicals and ROS are generated by the contracting skeletal muscles during physical exercise [26]. Interestingly, during moderate exercise, the exercise-induced ROS generation results in increased activity of enzymatic anti-oxidation mechanisms, which counter the pro-oxidation challenges [23,27]. In the present study, a bout of exercise of moderate intensity, in both studied groups at the third trimester, was followed by enhancement of pro-oxidation mechanisms (as indicated by the significant decrease of GSH concentrations) as well as of anti-oxidation mechanisms (as indicated by the significant increase in CAT activity and TAC in both studied groups).

Hyperglycemic environment is associated with enhanced pro-oxidation [5,6]. In women with GDM, ROS are increased, while mechanisms responsible to scavenge them are impaired [28]. This impairment has been attributed to decreased activity of enzymatic and non-enzymatic scavengers [29]. In the past, studies have shown decreased TAC and catalase activity in women with GDM, compared to pregnant women with uncomplicated pregnancies [29,30,31,32]. This is in accordance with decreased TAC and CAT activity in women with GDM as shown in the present study prior (baseline) to the bout of exercise. In a previous study in men with and without diabetes mellitus type 2 (T2DM), an exercise bout (cycling) of moderate intensity resulted in an increased anti-oxidation in controls but not in men with T2DM [33]. In the present study, the increase in the CAT activity immediately after the bout of exercise was significantly less pronounced in women with GDM compared to women with uncomplicated pregnancies. The deranged metabolic background may be at the origin of this less pronounced increase of CAT activity in women with GDM in comparison to women with uncomplicated pregnancies. Thus, acute aerobic exercise emerges as a possible diagnostic stimulation test of anti-oxidation capacity biomarkers useful in situations with defective anti-oxidation system as it has been shown in GDM pregnancies in the present study.

In addition, in the present study in women with GDM, pre-conceptional BMI and weight gain were negative predictors of ΔTAC (prior-to to one hour after exercise). In the past, we have shown that pre-pregnancy BMI in normal uncomplicated pregnancies is positively associated with maternal leptin concentrations and insulin resistance indices [34]. The latter is known to be positively associated with increased pro-oxidation status [35]. In addition, in women with excessive gestational weight gain, leptin concentrations were associated positively with pro-oxidation markers [36]. In the present study, in women with uncomplicated pregnancies the increase of ΔTAC and ΔCAT was not predicted by either maternal age or maternal pre-conceptional BMI or gestational weight gain or parity or history of GDM, indicating that physical exercise acts independently as an anti-oxidation beneficial factor.

Furthermore, in the present study, a bout of exercise of moderate intensity resulted in decreased mean UtA PI centiles in women with GDM but not in women with uncomplicated pregnancies. Regarding uncomplicated pregnancies, in the past, similarly to the findings of the present study, a bout of exercise of moderate intensity in pregnant women with uncomplicated pregnancies did not affect UtA PI centiles before and after exercise [37]. In fact, UtA PI was investigated in the present study because it is a marker of pregnancy health integrating and reflecting the state of different systems of maternal and fetal physiology, including utero-placental circulation and endothelial function, which are involved in high-risk pregnancies and more particularly GDM pregnancies [38,39].

In pregnancies with a well-regulated glucose-insulin axis, insulin signaling regulates oxidation status in endothelial cells resulting to relaxation and decreased vascular resistance. However, in GDM, increased insulin resistance leads to reduction of NO bioavailability and therefore, to vascular dysfunction explaining thus, the reported association of the latter with GDM [32,40,41]. Aerobic physical exercise decreases peripheral vascular resistance by neurohumoral, vascular, and structural adaptations, including alterations in the amount of vasodilator and vasoconstrictor molecules [42]. In non-pregnant patients, peripheral vascular resistance is reduced for up to 22 h after an aerobic exercise bout, with the greatest reduction observed in those with endothelial dysfunction [38]. This observation should be corroborated with the reduction, in the present study, in UtA PI centiles (representing UtA resistance) noted in the women with GDM and not in the women with uncomplicated pregnancies after the bout of aerobic exercise of moderate intensity. In the present study, in both women with GDM and with uncomplicated pregnancies the ΔUtA PI centiles were not predicted by either maternal age or maternal pre-pregnancy BMI or gestational weigh gain or parity or history of GDM or physical activity. This finding indicates that physical exercise acts independently upon uterine arteries resistance as a relaxation factor in women with GDM.

To our knowledge, this is the first study assessing the effect of a bout of moderate intensity aerobic exercise on oxidation capacity and UtA resistance (via measurement of UtA PI) in pregnant women with GDM and in women with uncomplicated pregnancies.

### 4.3. Limitations

A limitation of the study is the lack of evaluation of pro-oxidative markers. Evaluation of pro- and anti- oxidation markers would provide a more comprehensive depiction of the redox status changes after moderate exercise in uncomplicated pregnancies and pregnancies with GDM. Evaluation of mVO2max would have been helpful but it is not advised in pregnancy due to possible negative outcomes during the process of its estimation. The relatively small size of the study is also a limitation.

## 5. Conclusions

In conclusion, it appears that pregnancies complicated with GDM demonstrate a defective anti-oxidation system although they respond positively to the beneficial effect of a bout of aerobic exercise of moderate intensity during pregnancy, whereas pre-conceptional BMI and weight gain emerge as negative predictors of the anti-oxidation response. Of note, this bout of aerobic exercise of moderate intensity is followed by an improvement of resistance in uterine arteries of pregnant women with GDM, a phenomenon not observed in women with uncomplicated pregnancies. Thus, aerobic exercise of moderate intensity among its other proven beneficial effects, emerges as a useful therapeutic tool in pregnancies with GDM regarding the improvement of anti-oxidation capacity as well as UtA resistance. Further studies should investigate in a more detailed way this line of pathophysiology.

## Figures and Tables

**Table 1:** Anthropometric and pregnancy characteristics in women with GDM and women with uncomplicated pregnancies (control group). Self-reported exercise (light/moderate, at least for 30 min and 2 times/week). Quantitative variables are expressed in mean ± SD. Comparisons between groups were performed with Student’s *t*-test. Qualitative data are presented in percentages. Comparisons between groups were performed with Chi-square test.

Variable	GDM (N = 25)	Controls (N = 25)	*p*
**Maternal age in years** [mean (SD)]	32.4 (4.0)	30.4 (6.2)	0.222
**Gestational age in weeks** [mean (SD)]	32 (2.5)	31 (3.2)	0.186
**BMI before pregnancy** [mean (SD)]	27.3 (7.9)	25.1 (5.2)	0.220
**BMI during the study** [mean (SD)]	30 (5.7)	28.6 (5.0)	0.325
**Weight gain in kg** [mean (SD)]	8.2 (7.5)	10.7 (6.0)	0.261
**Parity**			
Para I [n (%)]	17 (68%)	15 (60%)	0.452
Para II [n (%)]	5 (20%)	6 (24%)	
Para III [n (%)]	2 (8%)	2 (8%)	
Para IV [n (%)]	0	2 (8%)	
Para VIII [n (%)]	1 (4%)	0	
**History of GDM** [n (%)]	4 (16%)	0	0.043
**Spontaneous conception** [n (%)]	25 (100%)	25 (100%)	1.0
**Exercise** [n (%)]	10 (40%)	10 (40%)	1.0

**Table 2:** Total antioxidant Capacity (TAC), Catalase activity (CAT) and Reduced Glutathione (GH) in women with GDM and in women with uncomplicated pregnancies (control group), prior-to, immediately after and one hour after the exercise.

	TAC (μmol/mL)		CAT (U/mg Hb)		GSH (μmol/g Hb)	
	GDM (N = 25)	Controls (N = 25)	GDM (N = 20)	Controls (N = 20)	GDM (N = 20)	Controls (N = 20)
**Prior to the exercise bout**	0.75 ± 0.09	0.81 ± 0.09	208.8 ± 53.2	274.5 ± 54.8 *	1.73 ± 1.08	1.91 ± 0.87
**Immediately** **after the exercise bout**	0.86 ± 0.11 ^†^	0.90 ± 0.11 ^†^	245.5 ± 63.3 ^†^	341.5 ± 59.3 ^†^	0.97 ± 0.62 ^†^	1.32 ± 0.48 ^†^
**1 h after the exercise bout**	0.75 ± 0.07	0.77 ± 0.11	241.4 ± 87.6	294.1 ± 51.2 *	1.27 ± 0.59 ^#^	1.54 ± 1.04 *^,#^

Data are expressed in mean ± SD. Statistical significance was set at *p* < 0.05. Data were analyzed using repeated measure ANOVA; Bonferroni correction was employed as post hoc test. The asterisk (*) indicates a statistically significant difference between the two groups (women with GDM compared to women with uncomplicated pregnancies (*p* < 0.05)). The dagger (†) indicated a statistically significant difference within the groups (between prior-to and immediately after the exercise (*p* < 0.001)) and the hashtag (#) indicates a statistically significant difference within the groups (between prior-to and one hour after the exercise (*p* < 0.05)).

**Table 3:** Uterine artery (UtA) pulsatility index (PI) centiles and Umbilical artery (UmA) pulsatility Index (PI) centiles in women with GDM and women with uncomplicated pregnancies (control group), prior-to, immediately after and one hour after the exercise bout.

	UtA PI Centiles		UmA PI Centiles	
	GDM Group (N = 25)	Control Group (N = 25)	GDM Group (N = 25)	Control Group (N = 25)
**Prior to the exercise bout**	42.8 ± 29.9	53.1 ± 30.3	47.0 ± 26.9	44.5± 29.4
**Immediately** **after the exercise bout**	26.6 ± 22.7 ^†^	56.7 ± 27.0 *	44.3 ± 30.7	43.5 ± 31.9
**1 h after the exercise bout**	26.8 ± 24.1 ^#^	50.9 ± 26.9 *	42.0 ± 34.5	52.7 ± 32.6

Data are expressed in mean ± SD. Statistical significance was set at *p* < 0.05. Data were analyzed using repeated measure ANOVA; Bonferroni correction was employed as post hoc test. The asterisk (*) indicates a statistically significant difference between the two groups (women with GDM compared to women with uncomplicated pregnancies (*p* < 0.01)). The dagger (†) indicated a statistically significant difference within the groups (between prior-to and immediately after the exercise (*p* < 0.05)), and the hashtag (#) indicates a statistically significant difference within the groups (between prior-to and one hour after the exercise (*p* < 0.01)).

## Data Availability

Not applicable.

## Supplementary Materials

The following supporting information can be downloaded at: https://www.mdpi.com/article/10.3390/diagnostics12071732/s1.
