# Supplementary figures and images for: The Effect of Physical Exercise on Oxidation Capacity and Utero-Placental Circulation in Pregnancies with Gestational Diabetes Mellitus and Uncomplicated Pregnancies, a Pilot Study
Source: Diagnostics (Basel). 2022 Jul 16;12(7):1732. doi: 10.3390/diagnostics12071732 (PMC9322693; doi:10.3390/diagnostics12071732)

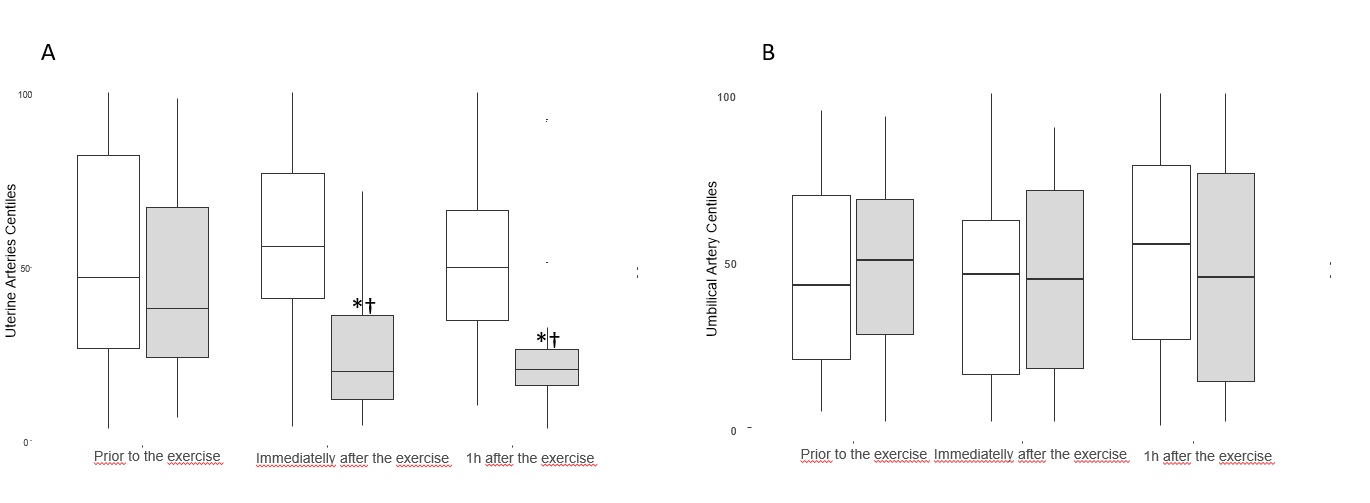

Supplement: Supplementary file 1 [file diagnostics-12-01732-s001.zip › diagnostics-1794357-supplementary.jpg]
